# "No God and no Norway": collective resource loss among members of Tamil NGO's in Norway during and after the last phase of the civil war in Sri Lanka

**DOI:** 10.1186/1752-4458-5-18

**Published:** 2011-08-17

**Authors:** Eugene Guribye

**Affiliations:** 1 Norwegian Institute of Public Health, Department of Mental Health, P.O. Box 4404 Nydalen, N-0403 Oslo

**Keywords:** Tamils, disaster, resource loss, coping, Norway, refugees

## Abstract

**Background:**

Studies on the mental health of refugees have tended to focus upon the impact of traumatic experiences in the country of origin, and acculturation processes in exile. The effects of crises in the country of origin on refugees living in exile have been little studied. This article examines how the final stages of the civil war in Sri Lanka in 2009 influenced members of pro-LTTE Tamil NGO's in Norway.

**Method:**

Ethnographic fieldwork methods were employed within Tamil NGO's in the two largest cities in Norway between November 2008 and June 2011.

**Results:**

The findings suggest that collective resources became severely drained as a result of the crisis, severely disrupting the fabric of social life. Public support from the majority community remained scarce throughout the crisis.

**Conclusions:**

The study suggests that there is a need for public support to exile groups indirectly affected by man-made crises in their country of origin.

## Background

In the final stages of the civil war between the Liberation Tigers of Tamil Eelam (LTTE) and the Government of Sri Lanka in 2009, hundreds of thousands of Tamil civilians became trapped in the line of fire inside an area roughly the same as New York City's Central Park. Tamils living in exile in Norway were powerless to prevent that tens of thousands of Tamil civilians were killed, many of them friends and family. This article draws on ethnographic fieldwork within pro-LTTE Tamil NGO's in Norway during the period, and examines how they were affected by the crisis in their home country while living in exile.

Research on the mental health of refugees has tended to focus on trauma induced by *pre migration *personal experiences in the home country (e.g., [1]), or on issues related to *post migration *acculturation and adaptation in the "host" society (e.g., [2,3]). However, when disasters occur, people originating from the affected area may find themselves affected in a number of ways although they are physically remote from the incident [4]. Nevertheless, the effects of crises that occur in the country of origin while refugees are comfortably situated in exile have been little studied. A few studies have focused on the impact of natural disasters on individual refugees [4-6]. The studies show that more than a year after the 2004 tsunami in Sri Lanka and Indonesia, refugees in exile who had lost friends or family in the disaster still had strong emotional reactions.

Previous research has suggested that within communities directly impacted by disasters, the social response may depend on the type of disaster [7,8]. While *natural *disasters may sometimes build a sense of community in which people help each other cope, *man-made *disasters may often destroy collective feelings of trust and security and create ambiguity and dissent in the affected communities. Furthermore, the loss of a sense of mastery and belief in a just world may aggravate stress and weaken people's stress resilience [9]. The result may be the development of subcultures of distress characterized by beliefs that may exacerbate and prolong the suffering of people attempting to cope with man-made disasters [10]. What is suggested here is that man-made disasters in the home country may potentially affect exiled refugees even more adversely than natural disasters. Yet there has been very little scientific focus on this.

### The civil war in Sri Lanka

The civil war in Sri Lanka has been a violent conflict going on for more than 25 years. Around 1 million Tamil refugees are currently estimated to live outside Sri Lanka. Experiencing discrimination from the Singhalese majority, the Tamil minority on the island raised a claim for a separate state in the 1970's. By the early 1980's the conflict eventually escalated into a full fledged civil war between Singhalese governmental forces and the Liberation Tigers of Tamil Eelam (LTTE). In 2002, a Norwegian-brokered cease fire agreement was signed, and the LTTE managed to establish a de facto Tamil state in the north east of Sri Lanka. After continued violations of the cease fire agreement on both sides, the government unilaterally abrogated the cease fire agreement in 2008, and the conflict escalated drastically again. As a result of an effective military campaign by the governmental forces, the remaining LTTE cadres along with 300 000 Tamil civilians became trapped within an area of a few square kilometres in the north east of the island.

On May 17^th ^2009, while Norwegians celebrated their National Day, the LTTE admitted military defeat, but their leadership was subsequently found dead. Tens of thousands of civilians were also killed in the last phase of the conflict, with estimates as high as 75 000 [11]. War crimes have been reported on both sides. Credible evidence has been presented of intentional shelling of civilians, hospitals and humanitarian operations by governmental forces, while the LTTE were reported to have caused intentional infliction of suffering on civilians [11,12]. After the victory, the Sri Lankan government interned both LTTE survivors and Tamil civilians in military controlled camps behind barbed wire, continuing to bar journalists and international aid workers. Reports of rape, abductions, general violence, malnourishment and outbreaks of disease within the camps nevertheless made it to international media [e.g. [13]] and have later been verified [12].

In March 2009, the Norwegian Institute of Public Health issued a report of concern to the Norwegian authorities, stating concern for the impact of the intensified civil war in Sri Lanka on the mental health and social integration of Tamil refugees in Norway. On background of this report of concern, it was necessary to properly document the impact of the disaster on the Tamil community.

### COR theory

Research has shown that although many victims of natural disasters are not necessarily directly exposed to life threatening or other traumatic experiences, they may suffer from adverse resource loss in the post disaster period. Resource loss may include scarcity of food, damage to property, and changes in personal characteristics (e.g., feelings of hopelessness) [14,15]. Traditional stress models, such as Lazarus and Folkman's stress-appraisal-coping theory, tend to define strain in terms of individuals' appraisals of events or situations as stressful [16]. In contrast, Conservation of resources theory (COR), as developed by Stevan Hobfoll [17,18] is based on the tenet that stress occurs when resources are lost or threatened or when individuals fail to replenish resources after significant investment. In principle, resource loss is more salient than resource gain, and loss may result in further loss cycles. As people lose resources, they become decreasingly capable of resisting threats to further resource loss. Resource loss in connection with man-made disasters such as war and terrorist attacks has been found to adversely affect individual functioning and cause psychological distress (e.g., [19-21]).

*Resources *are defined by Hobfoll [18] as valued objects, characteristics, conditions and energies which people strive to obtain and protect. In the current study, the main interest is in conditions and characteristics. These resources are particularly important since they provide access to other resources. C*onditions *such as social support or employment are resources that may lay the foundation for access to other resources. For instance, employment provides access to financial resources. *Cultural Characteristics *is used in this study to refer to cultural values and modes of organization that may indirectly provide access to collective resources. For instance, as will be shown, the common aspiration for a separate Tamil state in Sri Lanka has enabled Tamil exile communities to organize themselves effectively across caste, regional and religious distinctions. However, while cultural rituals may support the individual, repair rents in the social fabric, and re-establish the group during stressful times; massive upheavals may also result in cultural disintegration bereaving individuals of their protective cultural resources. This may have psychological consequences which may strongly affect people's lifestyles in the future [22].

In this theoretical framework, stress and coping are not merely linked to subjective cognitive processes, but also to the objective nature of people's environments, their resources. This provides a good starting point to explore how the disaster in the country of origin affected the Tamil social fabric of life in exile.

### The current study

According to COR theory, one would expect that even though Tamils living abroad were not directly exposed to life threatening experiences, the Vanni crisis would be likely to produce serious losses or threats within distinct resource categories. The current study focuses on the following research questions:

(1) In which ways do members of pro-LTTE Tamil NGO's in Norway experience loss of or threats to collective resources such as conditions and cultural characteristics during the crisis in their country of origin?

(2) In what ways do these collective resource threats and losses affect collective coping abilities?

## Method

### Approach

There was a need for an exploratory approach which would enable us to grasp the crisis from the perspective of the participants. Previous ethnographic fieldwork conducted among Tamils in Norway since 2006 had already established a level of trust between this researcher and Tamil informants, providing convenient access to the field during the last stages of the civil war in Sri Lanka. Between November 2008 and April 2011, participant observation was conducted among members of pro-LTTE Tamil NGO's in Norway with a starting point in the Tamil Resource and Counselling Centre (TRCC) and the Norwegian Tamil Health Organization (NTHO) in Oslo and Bergen. Note that although members of these organizations may be pro-LTTE, it does not necessarily indicate in any way that they constitute parts of the LTTE as such.

### Fieldwork setting

Norway is one of the most sparsely populated countries in Europe, rich in reserves of petroleum, natural gas, seafood, forests, and hydropower. The Scandinavian welfare model is designed to secure universal health care, subsidized higher education and a broad social security system for the country's population of 4.9 million people. According to the Human Development Index, Norway has consistently had one the highest human development and highest standard of living in the world during the last decade. Norwegians tend to view themselves as egalitarian people with a culture based on democratic principles, a trait which in the Norwegian public is often contrasted with the cultures of immigrants from non-western countries [23,24]. According to Statistics Norway as of January 2010 immigrants from 216 countries constitute a little over 11 percent of the total population. As of January 2007 there were 12 750 persons with Sri Lankan background in Norway, the majority Tamils. About 6000 of them live in Oslo and 1000 live in Bergen, the second largest city. A referendum conducted among Tamils in Norway in 2009 suggested that 99 percent of the voters endorsed the call for a sovereign Tamil state in Sri Lanka. According to the published results of the referendum, eighty percent of the eligible voters turned out for the referendum. This may imply that at least at the time, LTTE supporters may have formed a majority of the Tamil community [25].

Pro-LTTE NGO's such as TRCC and NTHO are important social arenas which provide Tamils with an opportunity to make culturally meaningful contributions for the well-being of Tamils in both the diaspora and in Sri Lanka [26]. TRCC aims to provide services and tuition which enables the integration of Tamils in the Norwegian society, while NTHO initiates health projects among Tamils in Sri Lanka and Norway. These NGO's will be further described in the Findings section. Note that unlike in many other countries, the LTTE is not an illegal organization in Norway.

### Participants and recruitment

Key informants consisted of 14 well-educated men and 6 well-educated women between 30-50 years old who had lived in the country for 10 years or more, and were members of Tamil NGO's in Norway. Most of these openly supported the LTTE, but to various degrees. Further recruitment of informants was characterised by a naturalistic "snowball" effect resulting from following flows of interaction among members of these organizations, and among these and other Tamils. Key informants recruited informants, who in turn recruited further informants and so forth. This process provided access to information from a broad spectrum of members of Tamil NGO's in Norway, including TRCC and NTHO, as well as members of The Economic Consultancy House (TECH), Tamil Rehabilitation Organization (TRO), Tamil Youth Organization (TYO), Tamil Women's Organization (TWO) and The Tamil Coordinating Committee (TCC). I also had the opportunity to talk to members of- and follow activities within the newly formed Transnational Government of Tamil Eelam (TGTE), and the National Council of Tamil Eelam (NCET). Participants were given oral information about the study and asked to give oral consent to participate in it.

### Procedure

Fieldwork consisted in participation in regular activities within the community (i.e. meetings, demonstrations, etc), and in-depth conversations with informants about their lived and subjective experiences during the crisis. Reports of the conflict in international media and content on Tamil websites and news blogs were reviewed to identify discourse patterns. The informants' responses to these reports and websites also came into focus. All key informants spoke Norwegian fluently, and helped interpret conversations in Tamil during the fieldwork. The role of the ethnographer in this context was that of a trusted resource person who aided in organizing interventions to help despairing Tamils, tried to raise awareness about the crisis in Sri Lanka in Norwegian media, and provided emotional and instrumental social support (e.g., independent Chief Election Commissioner for the 2010 elections for the Transnational Government of Tamil Eelam).

An analysis of data from previous ethnographic fieldwork among Tamils in Norway [27] enabled the identification of collective resources (*conditions *and *cultural characteristics*) within the Tamil community prior to the crisis. The next step was to investigate how these distinct resources were affected by the crisis. For instance, did the crisis result in changes in the way these Tamils organized themselves collectively? How important were the resources that were lost or threatened? How did the informants perceive these losses? And what meanings were constructed about the outcome of the crisis?

### Analysis

The analytical process may be described as a continuous pendulum between theoretical (re)assessment and empirical observation [28]. The general approach was partly built on methodological frameworks developed by Corbin & Strauss [29], and further developed by Emerson et al. [30]. Field notes were initially written up as fully as possible and later edited and organized into distinct themes, patterns, and variations related to the research questions. This process involved line by line categorization of paragraphs. Non-corresponding events were grouped in a separate category to investigate discrepancies between the theoretical framework and empirical observations. Field notes were then sorted according to these categories, and rearranged into new data sets by collecting together all data fragments that were related to each category. In this way, a sustained examination of the research questions was provided by linking together a variety of discrete observations. These categories were also assessed against the general understanding that derived from routinely participating in activities in the field.

## Findings

### Conditions

According to COR-theory, c*onditions *such as social support, employment and membership in organizations are resources that may lay the foundation for access to other resources. Collective conditions resources among Tamils in Norway prior to the crisis included the security of family members in the country of origin, integration in the Norwegian educational system and labour market, feeling general social support in the Norwegian society, membership in Tamil NGO's, and relatively stable social support networks.

#### Prior to the crisis

During the cease-fire agreement between 2002 and 2008, the security of family members in Sri Lanka was better than in many years, and many were able to travel back to their country of origin. Tamils in Norway had been marketed as "super immigrants" in the Norwegian public. They had been well-integrated in the labour market, had not depended upon welfare-services, had seldom been involved in crime, and the level of education among second generation Norwegian Tamils had been high [31-33]. Although they were reported to be less well socially integrated [24], the Norwegian facilitator role in Sri Lanka had provided Tamils with a general sense of social support in Norway. The Norwegian engagement in the peace negotiations had resulted in regular media coverage of the political development in Sri Lanka, and some Tamil community leaders had been able to meet and talk with Norwegian diplomats active in Sri Lanka.

In addition, through membership in Tamil NGO's in Norway, people were able to maintain meaningful activities related to the social conditions of Tamils in Norway, as well as in Sri Lanka [26,34]. NGO's such as the Norwegian Tamil Health Organization (NTHO), and the Tamil Rehabilitation Organization (TRO) allowed Tamils to contribute to health- and development projects primarily in LTTE-controlled areas of Sri Lanka. Donations to the Tamil Coordinating Committee (TCC), the international political branch of the LTTE, helped fund the activities of the LTTE in Sri Lanka. TCC was also instrumental in forming the Tamil Resource and Counselling Centre (TRCC). The centre focused on the integration of Tamils in the Norwegian society by providing courses and seminars on various aspects of the Norwegian society, as well as tuition services which gave Tamil children an improved chance of completing higher education and participating successfully in the Norwegian labour market. At the same time, mother-tongue tuition and cultural events were arranged at the centre with the aim of maintaining a sense of Tamil identity in exile [25].

Importantly, most of these Tamils indirectly regarded themselves as part of the LTTE, which was not merely perceived as a Tamil organization, but as the organization *of the Tamils*:

The media and the UN don't understand how Tamils think. How can they claim that the Tigers [LTTE] are preventing Tamil civilians from escaping from the battle zones? The international community fails to understand that the LTTE *is *the Tamils. They don't *want *to escape.

Although later investigations have concluded that the LTTE was in fact forcefully using civilians as a shield [11,12], the above statement by one informant during the Vanni crisis encapsulates the general sentiment within this part of the Tamil exile community at the time. In this context, the perception of being part of the LTTE did not imply participating on the battlefield, nor necessarily formal assignment of specific tasks within the organization. Rather, on a general level, it implied the acknowledgment of the LTTE as (the sole) representatives of the Tamils in the struggle for Tamil Eelam. This also enabled the mobilization of funds that according to some researchers prolonged the secessionist campaign in Sri Lanka [35].

#### Threats to the security of family members in the country of origin

The Vanni crisis represented a serious threat to the security of family members in the country of origin. No reporters, international aid workers or UN officials were allowed into the government's No Fire Zone where hundreds of thousands of Tamil civilians sought shelter. Without independent observers within the area, reliable information was mixed with rumours and propaganda. Accordingly, the faith of friends and relatives within the area remained uncertain. Many informants scoured through Tamil websites on a daily, if not hourly, basis looking for photos or reports of dead relatives or friends. In February 2009 a Tamil family on the west coast of Norway lost as many as 11 family members within the No Fire Zone in one day. According to later verified reports, the area was routinely shelled by governmental forces [12]. Even after the LTTE admitted defeat in May 2009, the security of family and friends in Sri Lanka continued to be threatened as civilians and surviving LTTE cadres were placed in internment camps behind barbed wire. Reports of violence, rape, disease and lack of food inside the camps generated great concern among informants.

#### Threats to integration in the Norwegian society

In September 2008, UN staff left the conflict zone out of security concerns, making many informants fear that the lack of international observers would lead to war crimes. By November 2008, Tamils in Norway started to mobilize resources to attract public attention to the situation in Sri Lanka. Hunger strikes, public rallies, torch processions and demonstrations were arranged. As time progressed and the situation in Sri Lanka became worse, some became engaged in political lobbying and attempted to mobilize the support of Norwegian politicians and Norwegian diplomats previously involved in the peace negotiations in Sri Lanka. Flyers were printed, Facebook groups were established, and email campaigns were organized to spread awareness about the situation in the general public. To an extent, by March 2009, public demonstrations became the new communal meeting point for many Tamils. This eventually gave rise to a new set of concerns within this part of the Tamil community.

In April 2009, many informants said they had heard reports from Vanni that around 400 LTTE soldiers had been killed by chemical weapons. Frustrated Tamils in Oslo blocked the traffic, struggled physically with the police, went on hunger strikes outside the Prime Minister's office, and demanded that former Special Envoy in Sri Lanka Erik Solheim should call for an emergency governmental meeting. A group of young Tamils also attacked the Sri Lankan embassy in Norway, and as a result, Norway was officially relieved of their facilitator role by the Sri Lankan government. Some Tamil parents feared that young Tamils would also start to emulate the acts of self-immolation of Tamils in other parts of the world. All these public outcries made many Tamils concerned with the effect of the situation on the long-standing public image of Tamils as "*super immigrants*" in Norway: "[T]he 12 000 Tamils in Norway have been regarded as a success story in terms of integration in the country; may this change now?" (Dagsnytt 18, NRK, April 14^th ^2009; translated from Norwegian). The fear was that Tamils would be labelled "*terrorists*" and "*full-time activists*":

"If the Tamils are so full of energy, they may just go home to their island and fight for a free Sri Lanka rather than going on a rampage in Norway and destroying public property. The scum is doing too well on public welfare. Treason to our nation!!!" (Participant in public web debate, VG http://vgd.no/index.php/samfunn/innvandring-rasisme-og-flerkultur/tema/1469316/innlegg/; translated from Norwegian.)

However, while members of Tamil NGO's were becoming concerned with the public image of Tamils in Norway, they observed that many younger Tamils had other concerns: "They say that it doesn't matter what reputation Tamils in Norway have, because if everybody dies in Sri Lanka, what is the point in living?". Reflecting on the general lack of public support at the time, a Tamil community leader stated that

"I always thought we were well integrated in the Norwegian society. But now I realize we are not. We have only been integrated in the labour marked. If we had truly been socially integrated, the whole nation would have supported us."

Another community leader expressed his sentiments this way: "For the first time I feel like a refugee in Norway", after more than 20 years in the country.

#### Loss of feeling of social support

As the crisis in their country of origin developed into a humanitarian disaster, many Tamils experienced a loss of the general feeling of social support they had experienced in Norway. The general lack of social integration among first generation Tamils implied that many of them had few ethnic Norwegian friends to turn to for emotional support: "I don't know who to turn to", a female informant said, "all other Tamils have the same problems and can't help me, and I don't have any good Norwegian friends". At the same time, public support remained relatively scarce. A point of major concern to many informants was that on February 3^rd ^2009, Norwegian diplomats and other co-chairs of Sri Lankan donor countries publically encouraged the LTTE to surrender. Many informants, on the other hand, had been expecting them to condemn attacks on civilian targets by governmental forces. Consequently, these official diplomatic statements were described by some Tamils as "a stab in the back by your best friend". One informant noted that "The statement was issued on the day before the National Celebration in Sri Lanka. Tamils regard this statement as a declaration of support of the government's warfare".

In January 2009, the humanitarian crisis in Gaza had engaged the Norwegian public to turn up in large numbers for public demonstrations. In contrast, during the Vanni-crisis, which was described by the Norwegian Minister of the Environment and International Development in a press release as "the greatest humanitarian crisis in the world", there was very little public engagement. Tamils found themselves marching in the streets virtually on their own, and when the Norwegian public did become engaged in the conflict, it tended to reinforce the sense of abandonment many of these Tamils felt at the time.

For instance, in March 2009, several international and national NGO's came together in Oslo to organize a torch procession for civilians in Sri Lanka. In an attempt at avoiding political controversy, LTTE symbols were banned from the procession. This provoked many young Tamils who clandestinely raised their Tiger flags while the arrangers vainly attempted to physically restrain them. Although the procession was organized to show the Tamil population that the Norwegian public had sympathy for their situation, Tamils completely outnumbered the few ethnic Norwegian participants in the procession. Out of around 1000 participants, around 40 Norwegians participated in the procession. Tamil participants looked around and commented that "there are not many Norwegians here". From the perspective of some Tamils, this attempted display of solidarity only served to reinforce the distance between themselves and the Norwegian public. Struggling to cope with his growing sense of alienation from the Norwegian society, one informant framed his experiences this way: "There is no God and no Norway".

#### Threats to social support networks

Many informants stated that they had enough problems coping on their own and had little support to provide for others during the Vanni crisis. At the same time, growing tensions and disarray within the Tamil community started to become visible. Tamil parents struggled to control Tamil youth who witnessed how their former role models in the first generation coped badly with the situation. Tamil community leaders were powerless to prevent Tamil youth from raising Tiger flags during the torch procession described above. Similarly, they could not prevent them from attacking the Sri Lankan embassy in Oslo. Other Tamil youth attempted to break out and generate support networks by becoming engaged in political lobbying. Their efforts to relieve the situation in Sri Lanka were largely conducted without involving members of the first generation of Tamil immigrants in Norway. "They will only object to our methods", one young Tamil explained, "They don't know what they're doing anymore".

After the defeat of the LTTE in May 2009, new political power structures also started to become visible within the global Tamil Diaspora, partly reflecting old fault lines within the LTTE. The result was that what many informants had formerly experienced as a united community became divided into fractions, and the previously well-functioning social support networks became fragmented. Former friends found themselves on opposing sides, some families were divided, and some former leaders withdrew from the TRCC in Oslo as a result of political disagreements. Many informants stated that this process contributed to aggravate their stress and make it even more difficult to find social support. Since this process was the result of certain changes in cultural characteristics, it will be described more fully below.

#### Cultural characteristics

*Cultural characteristics *refers to cultural values and modes of organization that may indirectly provide access to collective resources. Collective cultural characteristics resources among Tamils in Norway prior to the Vanni crisis included a collective cause that provided a common focal point in exile, the valuation of self-reliance, a sense of mastery, and belief in a just world.

#### Prior to the crisis

The common Tamil nationalist agenda had enabled Tamils to effectively organize themselves across caste, class, religious and regional differences. This had enabled Tamils access to other resources described elsewhere in this article, including the establishment of NGO's and social arenas in which resources such as social networks, knowledge and organizational skills had been collectively available.

These NGO's also enabled Tamils to maintain a sense of collective cultural identity in exile. In the context of state oppression of Tamils in Sri Lanka, this was considered an important collective resource among the informants. Tamil identity in exile was not merely related to Tamil language and culture, but also to the collective memory of traumatic experiences in the country of origin. Thus, Tamil parents considered it important for Tamil children to join Tamil NGO's where they could interact with other Tamil children and learn Tamil language and culture. Equally important, however, was the opportunity for their children to hear about the past experiences of other parents at the centre, as well as the ongoing experiences of Tamils in Sri Lanka. This collective memory of hardship and survival served as a motivational factor to succeed in exile, and to contribute towards improvement of social conditions in the country of origin.

These Tamils also valued self reliance and aspired to demand as little as possible from the Norwegian welfare system. Tamils had seldom been associated with crime or problems in the Norwegian media. Mistrust in governmental agencies seemed to have influenced Tamils throughout the civil war and the process of migration, making them generate their own social support networks in exile and rely on their own collective resources [36]. Among Tamils in Norway, public health services were for instance regarded with some scepticism. Tamils preferred to consult Tamil general practitioners with their problems, or relied on ambulant health services provided by Tamil NGO's [26]. As a result of this general tendency towards self-reliance, Tamils were generally not considered a burden on the Norwegian society, a factor which contributed to the marketing of Tamils as model immigrants in the country. This ascribed identity had provided Tamils with a collective sense of mastery and belief in a just world, and helped create opportunities for growth (e.g., employment, education, membership in voluntary organizations) in Norway.

#### Threats to the collective cause

When the LTTE was eventually defeated and its' leadership killed in May 2009, the collective cause seemed lost to many. With the goal of a Tamil state appearing to be lost, the effort to accumulate resources to reach towards it made little sense to many Tamils at the time. The initial sentiment, probably augmented by collective symptoms of depression, was that it was pointless to continue with the regular activities within the Tamil NGO's. On May 17^th^, the Norwegian National day, while Norwegians were busy celebrating their independence, confusing rumours were circulating about LTTE's defeat in Sri Lanka. On this day, my field notes read:

"[Name of informant] is dispirited; he has lost faith in everything, the international community, justice. Today he doesn't want to continue his work [in the Tamil NGO's]. 'I feel I have given everything I can to Norway, but Norway gave nothing back.' He fears that the community will dissolve, and that they no longer have anything to unite around.

Ten days later, the situation was even worse:

"[Name of informant] says that "I had anticipated that there would be a great deal of mental health problems among Tamils in this situation, but it is far worse than I had suspected." The uncertainty of [LTTE leader] Prabhakaran's fate (is he dead, is he alive?) has brought about a great strain on many. Many deny that he is dead, pointing towards the confusing statements that have been made in the press. At the same time, [name of informant] thinks that the denial in itself may be a sort of mental reaction, and that when it dawns on them that he [Prabhakaran] is actually dead, they will have an even bigger reaction. He repeats that all they have worked for the last 30 years is gone. (Excerpt from field notes, May 27^th ^2009.)

Among first generation Tamils, the responses to the military defeat were divided. Some were turning in sick-leaves from work, signalling a general lack of motivation which threatened to initiate a loss cycle. Others seemed to cling to a small fragment of hope, denying the death of LTTE leader Prabhakaran, and claiming that he would resurface as he had done previously. The whole situation contributed to create confusion and uncertainty within the community.

Almost immediately following the defeat of the LTTE, a ballot on the present validity of the political fundamentals of the Vaddukkoaddai Resolution of 1976 was organized within the Diaspora as a potentially unifying moment. According to the published results of the referendum, 80 percent of the eligible voters turned out for the referendum, and 99 percent of these endorsed the renewed call for a sovereign Tamil state in Sri Lanka [25]. Yet, new conflicts started to emerge concerning the way to proceed from the current situation. After the defeat of the LTTE in May 2009, Selvarasa Pathmanathan (known as KP) who was proclaimed as the new leader of the organization, called for an end of the armed struggle and a transition to political and diplomatic strategies. However, the so-called Castro-fraction of the organization hesitated to acknowledge KP as the new leader. Denying the death of Prabhakaran, they rather called for a continuation of the Diaspora funding of the LTTE [37]. Reflecting old fault lines within the LTTE, two central democratic organizations eventually emerged within the Tamil Diaspora. An unprecedented initiative was made within the KP fraction to form a democratic Transnational Government of Tamil Eelam (TGTE) to unite the Tamil Diaspora. Meanwhile, the Castro fraction formed the Global Tamil Forum (GTF), a transnational Tamil civil society movement amongst other things determined to:

"use all resources available to the Tamil Diaspora to establish the Tamil people's right to self determination and their right to re-establish their nationhood which was taken by force away from them by succeeding colonial powers including the Sri Lankan government" [38].

This political landscape contributed to dividing the community into several fractions which marked the emergence of what has been described as a paradigm shift within pro-LTTE parts of the Tamil Diaspora, from centrally organized activities to spontaneous and more unstructured events [39]. The general impression from long-term fieldwork within this community during the period was that individual power was very limited. The majority of Tamils involved in these new organizations were hard working idealists struggling to make an impact on the situation in Sri Lanka, but most of them had little or no previous experience with international politics. Hence, when viewed from the inside the power struggle had more of a quality of disempowerment. As a member of one of these new organizations suggested, "They all talk about a power struggle. But the truth is that none of us have any power at all".

The sudden lack of unity within the community was a source of concern for many, and some community leaders refused to take part in any political agendas until a return to unity had been ensured. Others felt pushed out of the community as a result of the new disputes. For whatever ambiguous reasons, the so-called "old guard" remained loyal to the tradition of the LTTE. They wanted to hold on to LTTE-related symbols and the goal of a separate Tamil state in Sri Lanka. More moderate Tamils found it unrealistic to lay claims to a separate state in the current setting, and felt the primary task was to rebuild the infrastructure in bombed villages in Sri Lanka. Some of the latter quickly became referred to as *Quislings*, traitors to the cause, and rumours circulated about candidly recorded phone conversations that served to reveal these Quislings within the community.

#### Loss of self reliance

Whereas self-reliance had been a major resource for the Tamil community prior to the crisis, it proved to be a terrible disadvantage during and after the crisis. One of the problems was that Tamils seemed to face something resembling a double bind: Overt support of the LTTE undermined their credibility within the international community, since the LTTE was an internationally condemned terrorist organization. Yet, overt criticism of the LTTE undermined the credibility of individuals within the Tamil community. While critisism of the LTTE had been moderate before the crisis, to oppose the LTTE *after *the loss was considered a blatant show of disrespect for the sacrifices of thousands of what these Tamils regarded as "freedom fighters" in the home country, while they themselves were living affluent lives in exile. Consequently, Norwegian journalists often stated to me that Tamils they had talked to appeared to be "brainwashed".

The crisis made it clear that the Tamil community was in desperate need of outside help. Initially there was a need for help from the international community to intervene in the conflict and save Tamil civilians trapped in the line of fire. After the end of the war, there was a need for help from the international community and the UN to secure the human rights of Tamil civilians inside the governmental internment camps. Eventually, there was a need for social support from the majority community, and for mental health services to help individuals, families and the community cope with grief over losses of friends and family. Although some welcomed the offer of support from public mental health services to cope with their grief, the general feeling among many informants was that "nobody will help the Tamils", At the same time, the crisis in the country of origin had a serious impact on community functioning. When the crisis intensified, most of the regular activities within the TRCC and other Tamil NGO's were cancelled. In addition, traditional rituals and celebrations were cancelled out of respect for the ordeals of Tamils in Sri Lanka. As a result, the fabric of social life within the Tamil community broke down and the community entered into something resembling a state of emergency. Without these collective social meeting points, social ties became weakened and the community became more fragmented. This created a need for outside support for resources that were normally available within the community.

#### Threats to a sense of mastery and belief in a just world

The loss of the war and the lack of international support contributed to a prevailing sense of defeatism and a lack of belief in a just world among the informants. In May 2009, after the LTTE had admitted defeat, a young informant perceived the situation in this way:

"We have just lost the war, our leaders are dead, our guns are captured, we have three hundred thousand civilians imprisoned in internment camps where a humanitarian crisis is evolving, the UN is actually financing the camps, and we have absolutely no international support whatsoever. And we think we can obtain a separate Tamil state *just like that*?"

Former UN spokesman in Colombo Gordon Weiss suggests that the LTTE's strategy had been to play out the "CNN effect" of a brutal siege of Tamil civilians on international public opinion [40]. Certainly, during the initial stages of the crisis, Tamil informants put their trust in the international community and the UN to intervene in the conflict. However, Weiss argues that the UN was hamstrung by the interests of some of its most powerful members, making it clear that there would be no resolution from the UN Security Council. A year after the war, the International Crisis Group called for an internal review of the UN's conduct during the war, suggesting that the organization was "close to complicit" in government atrocities [41]. This resonates with the general feeling of many informants during the time.

Even after the war, in June 2009, the main despair of many informants was that the UN helped finance the barbed wired military controlled internment camps in Sri Lanka amidst credible reports of rape, abductions, general violence, malnourishment and outbreaks of disease within the camps [12,13]: "If we cannot trust the UN, who can we trust?" Some of the community leaders were concerned that this loss of belief in a just world would result in a future renewal of the secessionist military campaign in Sri Lanka, rather than spur democratic engagement. Furthermore, they were concerned that it would result in an "integration trauma" among young Tamils in Norway. However, there were no clear signs that this was actually taking place on a large scale.

In May 2011 however, the UN published a report of the Secretary-General's Panel of experts on accountability in Sri Lanka [12] which spurred a renewed sense of hope within the Tamil community. The report suggested that there was credible evidence of war crimes and crimes against humanity on part of the Sri Lankan governmental forces during the final phase of the war. This made members of the newly founded Tamil political organizations more confident about their efforts, and that it would be possible to convince the international community that they had an obligation to hold the responsible parties accountable for their crimes during the war. By June 2011, a rekindled optimism was evident among members of some of these Tamil NGO's in Norway, and people who had briefly been divided along political fault lines were gradually starting to socialize again.

## Discussion

This paper has outlined how members of NGO's within a refugee community were collectively affected by a man-made crisis in their home country while living comfortably in exile. Described as "super immigrants" in the Norwegian society, these refugees experienced a radical breakdown of collective coping resources during the last phases of the civil war in their country of origin. This suggests that man-made crises in the country of origin may have a serious impact on refugee communities in exile, particularly on groups who are politically orientated.

A possible limitation of the study could be that it mainly focused on Tamils who supported LTTE, making the findings less valid for Tamils without this political orientation. However, the results of the referendum referred to above suggests that the separatist cause had a strong following among Norwegian Tamils at the time of the fieldwork [25]. The collective cause had provided these Tamils with a collective focal point in exile, and enabled them to organize themselves effectively across social distinctions. When this central collective resource was threatened, it led to threats to other collective resources as well. Yet, threats to collective resources such as the security of family members in the country of origin, and belief in a just world were not exclusively linked to politically orientated Tamils, and are likely to apply to other Tamils in the country as well.

Another limitation of the study is that correlations between resource loss and mental health problems among the participants have not been systematically investigated. A randomized controlled study could have provided us with a more detailed knowledge about relationships between crisis, resources and individual mental health. Previous research has demonstrated that serious resource loss in distinct resource categories predicts mental health problems [19-21]. Up until the Vanni crisis, studies on the mental health of Tamils in Norway have described a well coping community [34,42]. However, high rates of symptoms of depression, anxiety and bodily pains have been reported in a study among Tamils in Norway during the Vanni crisis [43]. The study shows that around 70% of the participants scored above clinical cutoff points for depression, while 35% of the participants scored above clinical cutoff points for anxiety. Importantly, around half of the participants in the cited study reported that their problems had an effect on their relationship with their children. Almost half of them (44%) had been on sick-leave during the period as a result of their mental health problems. It is likely that there is a connection between the serious mental health problems described in the above study and the threats to and loss of collective resources described in the current study. While this is beyond the scope of the current study, there is a need for more systematic studies of collective resource loss and collective mental health problems among refugees during crises in the country of origin.

Somasundaram [44] has reminded us that a broader, holistic perspective may become paramount in non-western "collectivist" societies which have traditionally been family or community oriented, and where individuals tend to become submerged in wider concerns. In contrast, western research and conceptualizations have been largely individualistic in orientation. In more collectivistic or co-operative societies, Somasundaram argues, we may need to go beyond the individual to the family, group, and community in order to more fully understand the individual, whether the concern is responses to stress or designing interventions. His arguments remain particularly valid in this context, since they are based on a study among Tamils internally displaced by the civil war in Sri Lanka. The study showed that family and community life had suffered due to deaths, separation and deprivations. Furthermore, relationships, trust, cohesion, beliefs and ethical values had declined, giving rise to conflict, bitterness, suspicion and sorrow. This process seems to be echoed in the current study among Tamils geographically far removed from the actual conflict zone, and reminds us of the importance of maintaining a methodological focus on individuals situated within a group or community context.

The impact of the disaster in Sri Lanka on the Tamil exile community far removed from the disaster seems to largely follow the same patterns as the impact of disasters on communities that experience it directly. Connections between a breakdown of the fabric of social life and levels of distress within the community following a disaster have been well documented. For instance, a study within six Guinean communities attacked by Sierra Leonan and Liberian RUF forces at the beginning of the millennium [45], shows that communities that disregarded community rituals and social support tended to have higher rates of distress. Communities that had concertedly resisted post-conflict social change had lower rates of distress. A study following the 2004 Tsunami in Tamil Nadu, India showed that resilient communities united among themselves, reduced communal conflicts, and maintained traditional rituals [46]. The current study, then, adds to the growing body of evidence suggesting a debilitating pattern of social response to disasters [7-10]. Fragmentation of communities following this type of disaster has been documented in a number of contexts. Erikson [7] notes that man-made disasters may force open fault lines that normally run silently through the structure of the larger community, splitting it into factions. De Vries [22] notes that at a group level a conservative response to severe stress may often take hold, involving a historical regression to idealized familiar conditions. Nationalism and fundamentalism may become means of survival to release individuals ideologically from an intolerable and unmanageable complexity. The present study suggests that this process may be augmented by loss of resources such as a sense of mastery, belief in a just world, and a general feeling of social support.

A major difference between the 2004 tsunami natural disaster and the 2009 man-made Vanni crisis was the response of the majority community towards the Norwegian Tamil community. The fact that many Norwegian tourists were also affected by the tsunami may have made the Norwegian society more sensitive to the needs of the Tamil community. A range of public interventions and financial support programmes were initiated at the time. For instance, bereaved Tamils were provided with financial support to travel back to Sri Lanka to bury their dead and generally be of help to their families and communities. They were provided with professional counselling, support hotlines for bereaved, temporary full time kindergarten, support groups, public meeting places, financial support, fund-raising for survivors on Sri Lanka, and memorial services [47].

In contrast, while there was a need for outside help during and after the man-made disaster in 2009, it was not forthcoming to the same extent. Norwegian diplomats were involved in a complex diplomatic process, but it largely occurred off the record, leaving many Tamils with the general impression that nothing was being done. In the end, the diplomatic negotiations seemed to have little effect on the outcome of the war. Furthermore, the sensitive political climate in Sri Lanka following the LTTE's defeat made it difficult for Norwegian Tamils to travel back to Sri Lanka at the time, and they were not offered financial support, at least not officially. Public interventions were far scarcer than during the Tsunami, even after the Norwegian Institute of Public Health issued a report of concern. Finally, the Norwegian population largely remained absent in Tamil public demonstrations. It is possible that this general lack of support within the majority community during the disaster was at least to some degree caused by the fact that the LTTE was an internationally banned terrorist organization, creating a difficult political and moral dilemma for local municipalities and the Norwegian government. The fact that Norway had been among the countries that did not ban the LTTE, and that Norwegian diplomats had been celebrated in the Norwegian public for their pivotal part in the previous peace negotiations in Sri Lanka, did not seem to alter this dilemma.

The study seems to support Hobfoll's suggestion that failure to gain resources after an investment may result in burnout, ill health and a detrimental motivational state [17]. An important observation is that what may constitute cultural characteristics resources in one context, may very well prove to become a disadvantage or a strain in another setting. The emphasis on self-reliance that had characterized the Tamil community prior to the crisis seemed to have contributed towards their so-called success in the Norwegian society. However this characteristic put them at a serious disadvantage during the crisis, when they were in desperate need of support but had no outside support network, or international political coalitions to rely on. This seems to echo the observation previously made that one may invest in coping resources in a problem that subsequently turns out to be a different one from the one initially envisioned. As a result, coping resources may be drained at exactly the moment when they are needed [26,48].

The study suggests that there is a need for more research on the effects of man-made disasters in the home country on the mental health of refugees in exile. There is reason to believe that man-made disasters in the country of origin may affect exile communities as severely as natural disasters such as the 2004 tsunami; and that they may have a need for help to cope with these types of disasters as well. Previous research has suggested that interventions should be early and intensive to counter act loss cycles [49]. Replenishing diminished resources may enhance the coping capacity within the community and reduce psychological distress. This may imply efforts to assist refugee communities in maintaining their fabric of social life. Models of disaster recovery are built around a return to normal life and stability after the disaster. However, as Couch & Mercuri [10] suggest, with man-made disasters, recovery must often take place in the midst of ongoing threats. Furthermore, recovery takes place within a social environment that, far from providing support, is often characterized by destructive social conflicts and the breakdown of community. Consequently, community building and conflict management may help people cope with the centrifugal forces set in motion by the disaster.

## Competing interests

The author declares that they have no competing interests.
